# Assessment of the Canadian Children’s Food and Beverage Advertising Initiative’s Uniform Nutrition Criteria for Restricting Children’s Food and Beverage Marketing in Canada

**DOI:** 10.3390/nu10070803

**Published:** 2018-06-22

**Authors:** Christine Mulligan, Marie-Ève Labonté, Laura Vergeer, Mary R. L’Abbé

**Affiliations:** 1 Department of Nutritional Sciences, Faculty of Medicine, University of Toronto, Toronto, ON M5S 3E2, Canada; christine.mulligan@mail.utoronto.ca (C.M.); laura.vergeer@mail.utoronto.ca (L.V.); 2 School of Nutrition & Institute of Nutrition and Functional Foods, Laval University, Québec City, QC G1V 0A6, Canada; marie-eve.labonte@fsaa.ulaval.ca

**Keywords:** nutrition, marketing, children, nutrient profiling

## Abstract

Imposing governmental restrictions on the marketing of unhealthy foods and beverages to children is a demanded policy action since in Canada, this remains self-regulated by the voluntary, industry-led Canadian Children’s Food and Beverage Advertising Initiative (CAI) whose participants pledge to only advertise products that satisfy its Uniform Nutrition Criteria to children. This study evaluated the stringency of this nutrient profiling (NP) model for restricting child-directed food and beverage marketing in Canada. Data was obtained from the University of Toronto Food Label Information Program (FLIP) 2013 database, providing nutritional information for 15,342 packaged products which were evaluated using the CAI Uniform Nutrition Criteria. Products with child-directed packaging and those from CAI participating companies were identified. Of the *n* = 15,231 products analyzed, 25.3% would be allowed and 57.2% would be restricted from being marketed to children according to the CAI Criteria. Additionally, 17.5% of products lacked criteria by which to evaluate them. Child-directed products represented 4.9% of all products; however, 74.4% of these would be restricted from being marketed to children under CAI standards. Products from CAI participating companies represented 14.0% of all products and 33.3% of child-directed products; 69.5% of which would be restricted from being marketed to children. These results indicate that if the CAI was mandatory and covered a broader range of advertising platforms, their Uniform Nutrition Criteria would be relatively stringent and could effectively restrict children’s marketing in Canada.

## 1. Introduction

Poor diet has been identified as a leading risk factor for death and disability among many Canadians [1]. The elevated prevalence of obesity in Canada contributes to a significant portion of this mortality risk [2]. Canadian children are not exempt from this trend, with the Heart and Stroke Foundation reporting that childhood obesity rates have tripled since 1979 [3], and the 2015 Canadian Community Health Survey showing that over one third of Canadian children currently have overweight or obesity [4].

A growing body of evidence shows that food and beverage advertising to children is playing a detrimental role in the childhood obesity crisis [5,6,7,8,9,10]. There is consensus in the literature that child-directed food products are typically energy dense and nutrient poor, and therefore do not represent ideal food choices for children [6,7,11,12]. Studies have shown that exposure to child targeted advertising results in increased consumption of less healthful foods by children and youth [6,7,10,13]. In response, government restrictions on unhealthy food and beverage marketing to children have been widely proposed as a means of protecting the health of this vulnerable population [3,5,7,8,13,14,15].

Currently in Canada, the only body overseeing food and beverage marketing to children is the Canadian Children’s Food and Beverage Advertising Initiative (CAI), introduced and monitored by Advertising Standards Canada as of April 2007 [16]. The CAI is an industry-led, voluntary code, currently holding commitments from 18 companies [16]. These companies have pledged to either eliminate advertising to audiences under 12 years of age entirely, or to advertise only products that meet the CAI’s Uniform Nutrition Criteria, a nutrient profiling (NP) model implemented by the CAI in December 2015 [16,17]. NP is the science of determining the degree of healthfulness of food products based on a set of nutritional criteria for a variety of nutrition-related public health purposes, such as restricting food and beverage marketing to children [18].

Since its development, the CAI has expanded its coverage to include media channels such as television, websites, video games and child-directed mobile media [16,17]. However, the CAI’s framework does not currently address all media used to market to children, such as child-directed product packaging, leaving manufacturers free to advertise to children on product packaging regardless of the nutritional quality of the product [16]. Studies have shown that voluntary, self-regulated initiatives such as the CAI have limited effectiveness in reducing children’s exposure to the promotion of unhealthy foods, further suggesting a need for government regulations on the marketing of unhealthy foods and beverages to children [15,19,20].

Therefore, the objective of this project was to evaluate the stringency of applying the CAI Uniform Nutrition Criteria for the purpose of restricting unhealthy food and beverage marketing to children in the Canadian context. Secondary objectives were to examine the extent to which products with child-directed packaging and products from CAI participating companies meet the CAI Uniform Nutrition Criteria.

## 2. Materials and Methods

### 2.1. Study Design

A cross-sectional analysis of the 2013 Canadian packaged food supply was conducted using nutritional data sourced from the University of Toronto Food Label Information Program 2013 database (FLIP 2013). Briefly, FLIP 2013 includes information on 15,342 unique packaged food products from the four top Canadian supermarket chains (Metro, Loblaw’s, Safeway, and Sobeys) which represent approximately 75% of the Canadian grocery retail market share. For each food product, FLIP 2013 includes information such as the nutritional values reported in the Nutrition Facts table (NFt), the list of ingredients, front-of-pack information (e.g., nutrient content claims and disease risk reduction claims), and company and brand information. Photos of products’ packaging are also available for analysis. The database is organized based on Schedule M of the *Food and Drug Regulations* (version current between 15 March 2012 and 13 December 2016) [21]. Schedule M includes 22 major food categories, and 153 subcategories. A more detailed description of FLIP 2013 is provided elsewhere [22].

### 2.2. Product Classification into CAI Uniform Nutrition Criteria Categories

First, products were classified into their appropriate CAI food categories and subcategories as described in the CAI White Paper [17]. Products that did not fall into any of the designated product categories were classified either as “Automatically Allowed” (products automatically allowed to be marketed to children; e.g., pure frozen fruits or vegetables), “Automatically Restricted” (products that are automatically restricted from being marketed to children; e.g., soft drinks), or as “No Criteria” (products for which the CAI has not established nutritional criteria; e.g., spices and sauces) [17].

Following the initial classification, a 10% random verification of the classifications into CAI food categories and subcategories was performed by a second evaluator, and a 95% agreement level was achieved. Categories with higher levels of disagreement (i.e., “Snacks”, “Dairy products and substitutes”, “Desserts”) were examined in depth, with any discrepancies resolved through consensus between researchers.

### 2.3. Product Evaluation Using the CAI Uniform Nutrition Criteria

Each product was evaluated based on the nutritional criteria outlined in the CAI White Paper for its respective subcategory [17]. For a product to “meet” the CAI Uniform Nutrition Criteria, it had to satisfy the criteria for both the “nutrients to limit” (e.g., sodium, total sugars) and the “nutrients to encourage” (e.g., whole grains, fibre and calcium). For example, to meet the Uniform Nutrition Criteria, a serving of cookies per stated size must contain: ≤150 kcal; ≤1.5 g of saturated fat; ≤190 mg of sodium; ≤10 g of sugars; ≥8 g of whole grain or 2 g of fiber, or ≥5% of the Daily Value (DV) of any essential nutrient (other than sodium) [17]. In this analysis, essential nutrients were considered to be vitamin C, vitamin A, calcium and iron, since these were the only nutrients available on all product NFts. Additionally, some of the CAI’s “nutrients to encourage” had to be estimated, such as whether the product contained the required 8 g of whole grain or ½ serving of Milk and Alternatives [17]. These could not be calculated directly from information on-package and instead were estimated from the ingredients lists. To increase the precision of our evaluations, these estimations were only conducted if they were the only remaining criterion that a product had to meet in order to satisfy the CAI criteria.

Products that required preparation (e.g., powdered puddings, dry pancake mix), were evaluated using the nutrient values for the “as consumed” version of the product in FLIP 2013; otherwise, “as purchased” values were used for all calculations.

### 2.4. Identification of Products with on-Package Marketing to Children

Products with child-directed packaging were identified within the FLIP 2013 database by analysing product packaging photos and were subsequently evaluated against the Uniform Nutrition Criteria. As detailed previously by Labonté et al. [23], criteria for identifying “child-directed” products were adapted from Colby et al. [24] and Elliott [25]. For a product to be considered “child-directed”, packaging had to include at least one of the following: (1) allusions to fun or play; (2) child-oriented lettering or graphics; (3) unconventional flavours, colours or shapes; (4) reference to toys, coupons, prizes or contests; (5) games; (6) children’s product lines (e.g., “mini” or “junior” product lines); or (7) characters appealing to children.

If a product’s packaging did not possess any aspects directed at children except for a character/image that was part of the product’s brand or company logo, it was not considered to be child-directed (e.g., Pita Break^TM^, Kraft© peanut butter and Pringles^®^ products were not considered to be child-directed since no other aspect of the packaging, other than their logo, would appeal to children). Branded characters (e.g., Kellogg’s Frosted Flakes’ Tony the Tiger©, Quaker’s Cap’n Crunch©), however, were considered as part of child-directed packaging. A second evaluator completed a 20% random verification of these classifications, and 97% agreement was achieved. Categories with higher levels of disagreement (i.e., “Bakery products”, “Desserts”, “Sugars and sweets”) were examined in further detail, with any discrepancies resolved through consensus between researchers.

### 2.5. Identification of Products from Companies Participating in the CAI

Despite child-directed product packaging not being included in the realm of the CAI [16], it is possible that companies who have committed to the CAI may be more likely to have child-directed products that meet the CAI criteria compared to products from companies who have not committed to the CAI, since their products would have to meet the CAI criteria to be advertised to children on platforms other than product packaging [16]. Given this rationale, products from companies who have pledged to be a part of the CAI were identified within the FLIP 2013 database. Products were considered to be from CAI participating companies if they were from any of the following 18 companies [16]: Campbell Company of Canada (Toronto, ON, Canada), Coca-Cola Ltd., (Atlanta, GA, USA), Danone Inc., (Paris, France), Ferrero Canada Ltd., (Brantford, ON, Canada), General Mills Canada Corporation, (Mississauga, ON, Canada), Hershey Canada Inc., (Mississauga, ON, Canada), Kellogg Canada Inc., (Mississauga, ON, Canada), Kraft Canada Inc., (Chicago, IL, USA), Maple Leaf Foods Inc., (Mississauga, ON, Canada), Mars Canada Inc., (Bolton, ON Canada), McDonald’s Restaurants of Canada Limited, (Toronto, ON, Canada), Mondelēz Canada, (Toronto, ON, Canada), Nestlé Canada Inc., (Toronto, ON, Canada), Parmalat Canada Inc., (Toronto, ON, Canada), PepsiCo Canada ULC, (Mississauga, ON, Canada), Post Foods Canada Inc., (Niagara Falls, ON, Canada), Unilever Canada Inc., (Toronto, ON, Canada), Weston Bakeries Limited, (Toronto, ON, Canada).

It is important to note that given the nature of the FLIP 2013 database, no products from McDonald’s Restaurants of Canada Limited were included. Products that did not come from any of the above companies were considered to be from CAI non-participating companies.

### 2.6. Statistical Analyses

The numbers and proportions of products that were considered to be “allowed” or “restricted” for marketing to children according to the CAI Uniform Nutrition Criteria were calculated overall and for each individual Schedule M category and subcategory. Analyses were repeated in the subset of products that were determined to have child-directed marketing on their packages. Additionally, analyses were repeated for all products and within the subset of products with child-directed packaging, separating products from CAI participating and non-participating companies. Given that many subcategories had few products from CAI participating companies, these analyses were completed only at the major category level. All descriptive statistics were calculated using SAS (version 9.3, Institute Inc., Cary, NC, USA).

## 3. Results

### 3.1. Evaluation of all Canadian Packaged Food and Beverage Products

In total, 15,231 products were included in the analyses following the exclusion of 111 products (*n* = 55 products which, based on Atwater calculations, had nutrient values in the NFt that were >20% different from the declared caloric values; *n* = 55 meal replacement drinks; and *n* = 1 Natural Health Product). Overall, 25.3% of products included in this sample would be allowed to be marketed to children; of these products, 21.5% met the Uniform Nutrition Criteria and 3.8% were considered to be automatically allowed (Table 1). Alternatively, 49.9% of products did not meet the CAI criteria, and 7.3% of products were automatically restricted, for a combined total of 57.2% of products that would be restricted from being marketed to children. There were missing data for an additional *n* = 7 products (0.05%), as values were missing for at least one of the nutrients that were required to determine whether a product would or would not meet the CAI criteria. However, in cases where values were missing for at least one of the tested nutrients but it did not affect the product’s evaluation (e.g., a “nutrient to encourage” had a missing value but the product already did not meet the “nutrients required to limit”), it was still possible to classify these products as ‘restricted’ from marketing to children. Therefore, such products were not considered as having missing data. A total of 17.5% of products could not be evaluated at all, as there were no relevant CAI criteria by which to evaluate them. 

#### 3.1.1. Categories with the Highest Proportion of Products that Would Be Allowed to Be Marketed to Children

Categories with the highest proportions of products (i.e., ≥50%) that would be allowed to be marketed to children included: “Eggs and Egg Substitutes” (75.0%); “Salads” (68.6%); “Dairy Products” (58.4%); “Nuts and Seeds” (57.3%); “Vegetables” (54.4%); “Potatoes, Sweet Potatoes and Yams” (52.9%); and “Fruit and Fruit Juices” (50.2%) (Table 1). For most categories, products would have to meet the CAI Uniform Nutrition Criteria in order to be marketed to children; however, in the “Vegetables” category, the majority of allowed products (96%; *n* = 435) were automatically allowed to be advertised to children. This included all products in the “Vegetables without Sauce” and “Lettuce and Sprouts” subcategories (Table A1). “Dairy Products” and “Fruit and Fruit Juices” also had a certain proportion of products (6.4% and 5.3%, respectively) that would not need to meet the CAI criteria in order to be marketed to children, and would be automatically allowed (Table 1).

#### 3.1.2. Categories with the Highest Proportion of Products that Would Be Restricted from Being Marketed to Children

In contrast, many categories had a large proportion of products that would be restricted from being marketed to children. Based on the CAI criteria, several categories consisted of >75% of products that would be restricted for marketing to children, including: “Beverages” (100.0%); “Dessert Toppings and Fillings” (100.0%); “Desserts” (92.7%); “Meat, Poultry, and Substitutes” (91.4%); “Soups” (84.9); “Marine, Fresh Water Animals” (81.6%); “Snacks” (79.0%); and “Bakery Products” (77.4%) (Table 1). All products in the “Beverages” and “Dessert Topping and Fillings” categories would be restricted from being marketed to children, since the products in these categories are automatically restricted under the CAI criteria. A large proportion of products in the “Sugars and Sweets” category (66.2%) were also automatically restricted from being marketed to children (Table 1).

#### 3.1.3. Categories for Which There Were no Relevant CAI Uniform Nutrition Criteria

Products for which there were no relevant CAI criteria included all products in the “Fats and Oils” and “Sauces, Dips, Gravies and Condiments”. Additionally, several products in the “Sugars and Sweets” (33.8%; e.g., jams, marmalades), “Vegetables” (39.9%; e.g., pickles, olives, vegetable purées), and “Miscellaneous Products” (50.7%; e.g., baking powder, seasonings, and spices) categories could not be evaluated due to the absence of relevant CAI criteria.

### 3.2. Evaluation of Canadian Packaged Food and Beverage Products with Child-Directed Packaging

#### 3.2.1. Categories with the Highest Proportion of Products with Child-Directed Packaging

As previously reported by Labonté et al. [23], sub-analyses showed that 4.9% (*n* = 747) of products analyzed had child-directed packaging (Table 2). The categories with the highest proportion of child-directed products were “Desserts” (17.4%, *n* = 144), “Sugars and Sweets” (9.6%, *n* = 72), “Bakery Products” (8.3%, *n* = 173), “Snacks” (6.8%, *n* = 54), “Dairy Products” (6.0%, *n* = 74) and “Dessert Toppings and Fillings” categories (6.0%, *n* = 7) (Table 2). Overall, these categories did not fare well by CAI standards, with at least 42% of all products (i.e., child-directed and those directed at a general audience) failing to meet the CAI Uniform Nutrition Criteria (Table 1). Only the “Dairy Products” category contained ≥50% of products considered to be allowed for children’s marketing according to the CAI (Table 1).

The child-directed products most commonly seen in Canada (i.e., *n* ≥ 40 child-directed products in an individual subcategory) belonged to one of the following subcategories: “Cookies, with or without coating/filling, graham wafers” (*n* = 84, 25.1% of “Cookies” subcategory); “Ice cream, frozen yogurt and sherbet” (*n* = 54, 13.9% of subcategory); “Chips, popcorn and extruded snacks” (*n* = 52, 9.3% of subcategory); “Juices, nectars and fruit drinks” (*n* = 45, 7.1% of subcategory); “Candies” (*n* = 45, 11.9% of subcategory) and “Dairy desserts, frozen” (*n* = 43, 23.0% of subcategory) (Table A2).

#### 3.2.2. Products with Child-Directed Packaging that Would Be Restricted from Being Marketed to Children

After evaluation using the CAI Uniform Nutrition Criteria, 74.4% of products with child-directed packaging did not meet the nutritional standards of the CAI and would be restricted from being marketed to children on other media included in the CAI framework (Table 2). At least 39.2% of child-directed products from each of the 16 major food categories containing products with child-directed packaging would not be allowed to be marketed to children, with 8 of these major categories having ≥75% of their child-directed products failing to meet the CAI criteria (Table 2). In the categories with the highest proportion of child-directed products, the vast majority of child-directed products would not be allowed to be marketed to children (i.e., 97.2% of “Desserts”, 86.1% of “Sugars and Sweets”, 73.4% of “Bakery Products”, 81.5% of “Snacks”, 100% of “Dessert Toppings and Fillings”; Table 2). The only exception was the “Dairy Products” category, which actually had the lowest proportion (39.2%) of child-directed products that would be restricted from marketing to children of any category containing child-directed products (Table 2).

### 3.3. Evaluation of Packaged Food Products from CAI Participating and Non-Participating Companies

#### 3.3.1. Evaluation of All Packaged Food and Beverage Products from CAI Participating and Non-Participating Companies

In total, 14.0% (*n* = 2131) products in FLIP 2013 were from CAI participating companies, and 86.0% (*n* = 13,093) were from non-participating companies (Table 3). Overall, the proportion of products from CAI participants that would be allowed to be marketed to children according to the CAI criteria was similar to that of non-participating companies (24.0% and 25.5%, respectively) (Table 3). 

#### 3.3.2. Evaluation of Products with Child-Directed Packaging from CAI Participating and Non-Participating Companies

When looking only at products with child-directed packaging, 33.3% (*n* = 249) were from CAI participating companies, and 66.7% (*n* = 498) were from non-participating companies (Table 4). A higher proportion of products with child-directed packaging from CAI participating companies (30.5%) would be allowed to be marketed to children according to the CAI compared to the proportion of child-directed products from non-participating companies that would be allowed to be marketed to children (19.9%) (Table 4).

## 4. Discussion

As mentioned, the voluntary, self-regulated, industry-led CAI is currently the only program in Canada that has been implemented to limit the marketing of unhealthy foods and beverages to children. However, the Government of Canada has recently committed to developing mandatory national regulations in this area. In October 2016, Health Canada published its Healthy Eating Strategy, a set of initiatives intended to make the “healthier choice the easier choice” for Canadians that specifically highlights the need to take action towards restricting the marketing of unhealthy foods and beverages to children [26]. Most notably, Senator Nancy Greene Raine proposed Bill S-228: *The Child Health Protection Act*, which as of February, 2018, has been passed in the Canadian Senate and is awaiting its third and final reading in the House of Commons, after which it will move to become Canadian law [27]. If passed, this bill would mandate that only “healthier” foods are marketed to Canadians under the age of 13 [27]. However, defining “healthier foods” requires an objective classification system, such as an NP model [14,18]. Health Canada has recently proposed a model for this purpose as part of a public consultation [28], but no final decisions have been made to date.

Therefore, the present study aimed to evaluate the application of the CAI Uniform Nutrition Criteria to the Canadian food supply for the purpose of restricting food and beverage marketing to children. Our results showed that only one quarter of packaged foods in Canada would be allowed to be marketed to children using this industry developed model. This can be compared to results from a recent study by our group in which we tested several NP models built by governmental or inter-governmental organizations for the purpose of restricting marketing to kids, using the same FLIP 2013 database [23]. In that earlier study, we found that the proportion of food products that would be allowed to be marketed to children varied considerably across four NP models (between 10–49% of foods) [23]. Results from the present study show that the CAI Uniform Nutrition Criteria would be stricter than both the World Health Organization’s Regional Office for Europe (WHO-EURO) NP model (30% of food products allowed) and the Food Standards Australia New Zealand Nutrient Profiling Scoring Criterion (FSANZ-NPSC) (49%), but less strict than the original Pan American Health Organization (PAHO, Washington, DC, USA) NP model (16%) and a modified version of the PAHO NP model (10%) [23]. It is worth acknowledging, however, that for the comparison of these results with the results of Labonté et al., (2017), products with “No Criteria” would have to be excluded from the assessments using the 4 authoritative-based NP models so that only products that could be evaluated using all models would be included in that analysis. In another study, Ni Mhurchu and colleagues tested the New Zealand Ministry of Health Food and Beverage Classification System (FBCS), Health Star Rating (HSR) system and the WHO-EURO NP model on a sample of over 13,000 packaged products in New Zealand [29]. Their results showed that each of these models would permit roughly a third (29–41%) of products to be marketed to children, more than the CAI [29]. In combination, these findings suggest that the CAI Uniform Nutrition Criteria would be more restrictive than these alternative NP models, but less strict than the PAHO model. Given that the NP model to restrict the marketing of unhealthy foods to children in Canada has not yet been finalized, a comparison of the CAI to this proposed NP model would be preliminary and outside the scope of this study. Future research will be needed to evaluate the potential impact of such a policy once the specific regulations have been published.

The current study suggests that the CAI could be an effective model to restrict the marketing of foods that are widely considered to be of lower nutritional quality. For example, it completely restricts soft drinks and confectionary, which are often noted as being poor dietary choices for children [14,30]. Products in other categories such as “Desserts”, “Snacks” and some “Bakery products” which should be consumed only occasionally [30] are also largely found to have a nutritional profile that does not meet the CAI requirements. On the other hand, many “Vegetables” and “Nuts and Seeds” were found to be allowed for marketing to children according to the CAI. Generally, the way in which the CAI evaluates different types of products is in line with healthy dietary patterns, such as the proposed recommendations for the new Canada’s Food Guide [31].

Since approximately three quarters of products that are currently marketed to children on their packaging would not meet the CAI criteria with their current nutritional composition, adding product packaging in the realm of the CAI would be an effective way to increase the CAI’s impact on limiting the marketing of unhealthy foods to children. These results also highlight the importance of ensuring that on-package marketing to children is included in any system designed to restrict marketing to children since most products marketed in this way were found to be unhealthy. Applying the CAI as a mandatory system could also motivate product reformulation, particularly for those manufacturers targeting children. These findings reiterate the stringency of this NP model, while also reinforcing the need for a policy to restrict food and beverage marketing to children, since this study and others [32,33,34] show that child-directed products tend to be from “less healthy” food categories, and also less healthy than the overall food supply.

Analysis showed that most products with child-directed packaging were from non-participating companies, and a higher proportion of child-directed products from CAI participating companies met the CAI criteria compared to products from non-participating companies. In combination, these findings suggest that participating companies’ pledges to eliminate or only advertise healthier products to children in other media may be carrying over to the packaged food environment. However, products from CAI participating companies make up less than one-fifth of the packaged food supply and over two-thirds of their child-directed products still fail to satisfy the CAI’s nutritional standards. These results highlight the limitations of voluntary approach to restricting food and beverage marketing to children, and support the need for mandatory regulations with a broad coverage of potential marketing platforms.

A major strength of the CAI NP model is the consideration of both positive (e.g., fiber) and negative (e.g., sodium) nutrients in its nutritional criteria, to give a more wholesome evaluation of the nutritional quality of the product. While researchers have criticized such a system as it may encourage the addition of ‘positive nutrients’ (e.g., fiber, protein isolates) [35] or discretionary addition of vitamins and minerals [36] rather than reductions in nutrients to limit (e.g., sodium), the CAI criteria require that a product satisfy both the positive and negative criteria separately, in order to be allowed to be marketed to children. As such, a higher level of stringency is maintained as compared with other models in which the positive nutrients can ‘balance out’ the negative nutrients and raise a product’s NP score [37,38]. Importantly, the CAI NP model is already in use by many major food companies and one large restaurant chain [16], which speaks to the acceptability of this model by industry stakeholders.

As with any NP model, the CAI has its limitations. Nearly one fifth of products had no criteria by which to be evaluated, reducing the scope of these criteria. However, these products were from food categories with very few child-directed products, indicating that including these products in their criteria would not likely have a critical impact on restricting children’s products. Additionally, the CAI NP model uses multiple food categories that are not always clearly defined, making product classification ambiguous (e.g., “Other Snacks”) [17]. It is generally accepted that having fewer food categories is the favored approach (i.e., the Ofcom NP model [38]) since little classification is necessary, and all products are evaluated by more consistent criteria [39,40]. Worth noting is that WHO Euro uses a category based NP model; however, its adoption is not yet widespread throughout Europe [41]. Should the CAI be considered, a modification of the number of food categories could be warranted; otherwise, clearer guidelines would be necessary in order to facilitate consistent product classification and evaluation. Additionally, clear guidelines on which nutrients are to be considered as “essential nutrients” in the CAI’s “nutrients to encourage” would be necessary. Should the CAI Uniform Nutrition Criteria be used as the NP model to fulfill the requirements of Bill S-228 as has been proposed [27], other existing limitations of the current CAI structure, such as its voluntary nature and limited coverage of marketing techniques and participating companies would no longer apply.

A major strength of this study was the use of a large, highly representative sample of food and beverage products available to Canadians, and presents the first analysis of the potential impact of scaling up a voluntary NP model for use as a mandatory NP model in Canada. Nonetheless, this work was not without its limitations. Firstly, it is important to note that the FLIP 2013 database does not contain any fresh, unpackaged foods. This is important to take into account when interpreting our results, since fresh products such as fruits and vegetables would be “automatically allowed” by the CAI and are not included in the analysis and therefore our results may portray the CAI as more restrictive than if fresh products were included. Additionally, as described previously, some of the CAI’s “nutrients to encourage” had to be estimated using the available NFt information and therefore may not represent the true composition of these components for certain products.

## 5. Conclusions

The CAI Uniform Nutrition Criteria offer a relatively stringent approach to restricting the marketing of unhealthy foods and beverages to Canadian children, but the voluntary nature of this approach and its failure to address certain marketing platforms (e.g., product packaging), is largely insufficient and urgent regulatory action in this area—such as the implementation of Bill-S228—is needed. Legislation mandating adherence to the CAI NP model for products marketed across all child-directed settings and communication channels would, however, likely prove effective in promoting a children’s food environment in line with dietary guidelines and national nutrition goals [27,31,42].

## Figures and Tables

**Table 1:** Evaluation of Canadian packaged food and beverage products ^1^ according to the Canadian Children’s Food and Beverage Advertising Initiative’s (CAI) Uniform Nutrition Criteria ^2^, presented overall and by major food category ^3^.

Schedule M Category ^3^					CAI Uniform Nutrition Criteria ^2^													
					Allowed						Restricted							
	Total Products Analyzed		Missing Data ^4^		Meets CAI Criteria ^2^		Automatically Allowed ^2^		Total Allowed		Does Not Meet CAI Criteria ^2^		Automatically Restricted ^2^		Total Restricted		No Criteria ^2^	
	*n*	% ^5^	*n*	% ^6^	*n*	% ^6^	*n*	% ^6^	*n*	% ^6^	*n*	% ^6^	*n*	% ^6^	*n*	% ^6^	*n*	% ^6^
TOTAL	15,231	100.0	7	0.0	3278	21.5	572	3.8	3850	25.3	7606	49.9	1105	7.3	8711	57.2	2663	17.5
1. Bakery Products	2084	13.7	2	0.1	470	22.6	0	0.0	470	22.6	1612	77.4	0	0.0	1612	77.4	0	0.0
2. Beverages (soft drinks, iced teas, coffees)	482	3.2	0	0.0	0	0.0	0	0.0	0	0.0	0	0.0	482	100.0	482	100.0	0	0.0
3. Cereals, other grain products	988	6.5	1	0.1	332	33.6	0	0.0	332	33.6	592	59.9	0	0.0	592	59.9	63	6.4
4. Dairy products, substitutes	1240	8.1	1	0.1	645	52.0	79	6.4	724	58.4	515	41.5	0	0.0	515	41.5	0	0.0
5. Desserts (Dairy and non-Dairy)	827	5.4	0	0.0	59	7.1	0	0.0	59	7.1	767	92.7	0	0.0	767	92.7	1	0.1
6. Dessert toppings, fillings	116	0.8	0	0.0	0	0.0	0	0.0	0	0.0	0	0.0	116	100.0	116	100.0	0	0.0
7. Eggs, egg substitutes	56	0.4	0	0.0	42	75.0	0	0.0	42	75.0	14	25.0	0	0.0	14	25.0	0	0.0
8. Fats, oils	535	3.5	0	0.0	0	0.0	0	0.0	0	0.0	0	0.0	0	0.0	0	0.0	535	100.0
9. Marine, fresh water animals	440	2.9	0	0.0	81	18.4	0	0.0	81	18.4	359	81.6	0	0.0	359	81.6	0	0.0
10. Fruit, fruit juices	1089	7.1	0	0.0	489	44.9	58	5.3	547	50.2	528	48.5	0	0.0	528	48.5	14	1.3
11. Legumes	180	1.2	0	0.0	89	49.4	0	0.0	89	49.4	91	50.6	0	0.0	91	50.6	0	0.0
12. Meat, poultry, their products, substitutes	895	5.9	0	0.0	77	8.6	0	0.0	77	8.6	818	91.4	0	0.0	818	91.4	0	0.0
13. Miscellaneous	450	3.0	0	0.0	18	4.0	0	0.0	18	4.0	193	42.9	11	2.4	204	45.3	228	50.7
14. Combination Dishes	1357	8.9	3	0.2	473	34.9	0	0.0	473	34.9	881	64.9	0	0.0	881	64.9	0	0.0
15. Nuts, seeds	220	1.4	0	0.0	126	57.3	0	0.0	126	57.3	87	39.5	0	0.0	87	39.5	7	3.2
16. Potatoes, sweet potatoes, yams	140	0.9	0	0.0	74	52.9	0	0.0	74	52.9	66	47.1	0	0.0	66	47.1	0	0.0
17. Salads	70	0.5	0	0.0	48	68.6	0	0.0	48	68.6	22	31.4	0	0.0	22	31.4	0	0.0
18. Sauces, dips, gravies, condiments	1229	8.1	0	0.0	0	0.0	0	0.0	0	0.0	0	0.0	0	0.0	0	0.0	1229	100.0
19. Snacks (Chips, pretzels, meat or nut snacks)	794	5.2	0	0.0	167	21.0	0	0.0	167	21.0	627	79.0	0	0.0	627	79.0	0	0.0
20. Soups	456	3.0	0	0.0	69	15.1	0	0.0	69	15.1	387	84.9	0	0.0	387	84.9	0	0.0
21. Sugars, sweets	749	4.9	0	0.0	0	0.0	0	0.0	0	0.0	0	0.0	496	66.2	496	66.2	253	33.8
22. Vegetables	834	5.5	0	0.0	19	2.3	435	52.2	454	54.4	47	5.6	0	0.0	47	5.6	333	39.9

^1^ Packaged food products are from the Food Label Information Program (FLIP) 2013 database, as described in [22]. ^2^ Canadian Children’s Food and Beverage Advertising Initiative’s (CAI) Uniform Nutrition Criteria are defined in: Advertising Standards Canada. *Canadian Children’s Food and Beverage Advertising Initiative*; Uniform Nutrition Criteria White Paper; September 2014 [17]. ^3^ Food categories are defined in Schedule M of the Food and Drug Regulations (C.R.C., c. 870), Sections B.01.001, B.01.002A and D.01.001, Government of Canada, Health Canada [21]. ^4^ Missing data indicates products which were evaluated, but values were missing for at least one of the tested nutrients that were required to determine whether a product would or would not meet the CAI criteria. However, in cases where values were missing for at least one of the tested nutrients but it did not affect the product’s evaluation (e.g., a “nutrient to encourage” had a missing value but the product already did not meet the “nutrients required to limit”), it was still possible to classify these products as or ‘restricted’ from marketing to children. Therefore, such products were not considered as having missing data. ^5^ Percentage of total products (i.e., out of *n* = 15,231 products). ^6^ Percentage of total products in that food category.

**Table 2:** Proportion of packaged food and beverage products ^1^ with child-directed packaging ^2^ that would be allowed to be advertised to children according to the CAI’s Uniform Nutrition Criteria ^3^, presented overall and by major food category ^4^.

	All Packaged Foods ^1^		Packaged Foods with Child-Directed Packaging ^2^							
Schedule M Category ^4^	Total Products Analyzed		Total Products Analyzed		Total Allowed ^3^		Total Restricted ^3^		No CAI Criteria ^3^	
	*n*	% ^5^	*n*	% ^6^	*n*	% ^7^	*n*	% ^7^	*n*	% ^7^
TOTAL	15,231	100.0	747	4.9	175	23.4	556	74.4	16	2.1
1. Bakery Products	2084	13.7	173	8.3	46	26.6	127	73.4	0	0.0
2. Beverages (soft drinks, iced teas, coffees)	482	3.2	11	2.3	0	0.0	11	100.0	0	0.0
3. Cereals, other grain products	988	6.5	51	5.2	25	49.0	26	51.0	0	0.0
4. Dairy products, substitutes	1240	8.1	74	6.0	45	60.8	29	39.2	0	0.0
5. Desserts (Dairy and non-Dairy)	827	5.4	144	17.4	4	2.8	140	97.2	0	0.0
6. Dessert toppings, fillings	116	0.8	7	6.0	0	0.0	7	100.0	0	0.0
7. Eggs, egg substitutes	56	0.4	0	0.0	N/A		N/A		N/A	
8. Fats, oils	535	3.5	0	0.0	N/A		N/A		N/A	
9. Marine, fresh water animals	440	2.9	2	0.5	0	0.0	2	100.0	0	0.0
10. Fruit, fruit juices	1089	7.1	58	5.3	18	31.0	40	69.0	0	0.0
11. Legumes	180	1.2	0	0.0	N/A		N/A		N/A	
12. Meat, poultry, their products, substitutes	895	5.9	4	0.4	1	25.0	3	75.0	0	0.0
13. Miscellaneous	450	3.0	14	3.1	0	0.0	8	57.1	6	42.9
14. Combination Dishes	1357	8.9	69	5.1	20	29.0	49	71.0	0	0.0
15. Nuts, seeds	220	1.4	9	4.1	4	44.4	5	55.6	0	0.0
16. Potatoes, sweet potatoes, yams	140	0.9	4	2.9	2	50.0	2	50.0	0	0.0
17. Salads	70	0.5	0	0.0	N/A		N/A		N/A	
18. Sauces, dips, gravies, condiments	1229	8.1	0	0.0	N/A		N/A		N/A	
19. Snacks (Chips, pretzels, meat or nut snacks)	794	5.2	54	6.8	10	18.5	44	81.5	0	0.0
20. Soups	456	3.0	1	0.2	0	0.0	1	100.0	0	0.0
21. Sugars, sweets	749	4.9	72	9.6	0	0.0	62	86.1	10	13.9
22. Vegetables	834	5.5	0	0.0	N/A		N/A		N/A	

^1^ Packaged food products are from the Food Label Information Program (FLIP) 2013 database, as described in [22]. ^2^ Products with child-directed packaging were identified using criteria based on previous publications [23]. ^3^ Canadian Children’s Food and Beverage Advertising Initiative’s (CAI) Uniform Nutrition Criteria are defined in: Advertising Standards Canada. *Canadian Children’s Food and Beverage Advertising Initiative*; Uniform Nutrition Criteria White Paper; September 2014 [17]. ^4^ Food categories are defined in Schedule M of the Food and Drug Regulations (C.R.C., c. 870), Sections B.01.001, B.01.002A and D.01.001, Government of Canada, Health Canada [21]. ^5^ Percentage of total products (i.e., out of *n* = 15,231 products). ^6^ Percentage of total products in that food category. ^7^ Percentage of products with child-directed packaging in that food category.

**Table 3:** Proportion of packaged food and beverage products ^1^ offered by CAI participating and non-participating companies ^2^ that would be allowed to be advertised to children according to the CAI’s Uniform Nutrition Criteria ^3^, presented overall and by food category ^4^.

Schedule M Category ^4^	All Packaged Foods ^1^				Packaged Foods from CAI Participating Companies ^2^								Packaged Foods from Non-Participating Companies							
	Total Products Analyzed		Missing Data ^5^		Total Products Analyzed		Total Allowed ^3^		Total Restricted ^3^		No CAI Criteria ^3^		Total Products Analyzed		Total Allowed ^3^		Total Restricted ^3^		No CAI Criteria ^3^	
	*n*	% ^6^	*n*	% ^7^	*n*	% ^7^	*n*	% ^8^	*n*	% ^8^	*n*	% ^8^	*n*	% ^7^	*n*	% ^9^	*n*	% ^9^	*n*	% ^9^
TOTAL	15,231	100.0	7	0.0	2131	14.0	511	24.0	1431	67.2	189	8.9	13,093	86.0	3339	25.5	7280	55.6	2474	18.9
1. Bakery Products	2084	13.7	2	0.1	358	17.2	87	24.3	271	75.7	0	0.0	1724	82.7	383	22.2	1341	77.8	0	0.0
2. Beverages (soft drinks, iced teas, coffees)	482	3.2	0	0.0	172	35.7	0	0.0	172	100.0	0	0.0	310	64.3	0	0.0	310	100.0	0	0.0
3. Cereals, other grain products	988	6.5	1	0.1	122	12.3	76	62.3	44	36.1	2	1.6	865	87.6	256	29.6	548	63.4	61	7.1
4. Dairy products, substitutes	1240	8.1	1	0.1	281	22.7	164	58.4	117	41.6	0	0.0	958	77.3	560	58.5	398	41.5	0	0.0
5. Desserts (Dairy and non-Dairy)	827	5.4	0	0.0	202	24.4	4	2.0	198	98.0	0	0.0	625	75.6	55	8.8	569	91.0	1	0.2
6. Dessert toppings, fillings	116	0.8	0	0.0	14	12.1	0	0.0	14	100.0	0	0.0	102	87.9	0	0.0	102	100.0	0	0.0
7. Eggs, egg substitutes	56	0.4	0	0.0	0	0.0	N/A		N/A		N/A		56	100.0	42	75.0	14	25.0	0	0.0
8. Fats, oils	535	3.5	0	0.0	93	17.4	0	0.0	0	0.0	93	100.0	442	82.6	0	0.0	0	0.0	442	100.0
9. Marine, fresh water animals	440	2.9	0	0.0	0	0.0	N/A			N/A	N/A		440	100.0	81	18.4	359	81.6	0	0.0
10. Fruit, fruit juices	1089	7.1	0	0.0	92	8.4	34	37.0	58	63.0	0	0.0	997	91.6	513	51.5	470	47.1	14	1.4
11. Legumes	180	1.2	0	0.0	1	0.6	1	100.0	0	0.0	0	0.0	179	99.4	88	49.2	91	50.8	0	0.0
12. Meat, poultry, their products, substitutes	895	5.9	0	0.0	62	6.9	4	6.5	58	93.5	0	0.0	833	93.1	73	8.8	760	91.2	0	0.0
13. Miscellaneous	450	3.0	0	0.0	74	16.4	3	4.1	51	68.9	20	27.0	376	83.6	15	4.0	153	40.7	208	55.3
14. Combination Dishes	1357	8.9	3	0.2	227	16.7	58	25.6	169	74.4	0	0.0	1127	83.1	415	36.8	712	63.2	0	0.0
15. Nuts, seeds	220	1.4	0	0.0	16	7.3	0	0.0	16	100.0	0	0.0	204	92.7	126	61.8	71	34.8	7	3.4
16. Potatoes, sweet potatoes, yams	140	0.9	0	0.0	12	8.6	2	16.7	10	83.3	0	0.0	128	91.4	72	56.3	56	43.8	0	0.0
17. Salads	70	0.5	0	0.0	1	1.4	0	0.0	1	100.0	0	0.0	69	98.6	48	69.6	21	30.4	0	0.0
18. Sauces, dips, gravies, condiments	1229	8.1	0	0.0	71	5.8	0	0.0	0	0.0	71	100.0	1158	94.2	0	0.0	0	0.0	1158	100.0
19. Snacks (Chips, pretzels, meat or nut snacks)	794	5.2	0	0.0	25	3.1	5	20.0	20	80.0	0	0.0	769	96.9	162	21.1	607	78.9	0	0.0
20. Soups	456	3.0	0	0.0	171	37.5	37	21.6	134	78.4	0	0.0	285	62.5	32	11.2	253	88.8	0	0.0
21. Sugars, sweets	749	4.9	0	0.0	97	13.0	0	0.0	95	97.9	2	2.1	652	87.0	0	0.0	401	61.5	251	38.5
22. Vegetables	834	5.5	0	0.0	40	4.8	36	90.0	3	7.5	1	2.5	794	95.2	418	52.6	44	5.5	332	41.8

^1^ Packaged food products are from the Food Label Information Program (FLIP) 2013 database, as described in [22]. ^2^ Companies Participating in the CAI, as Defined by the CAI Core Principles, October 2017 [16]. ^3^ Canadian Children’s Food and Beverage Advertising Initiative’s (CAI) Uniform Nutrition Criteria are defined in: Advertising Standards Canada. *Canadian Children’s Food and Beverage Advertising Initiative*; Uniform Nutrition Criteria White Paper; September 2014 [17]. ^4^ Food categories are defined in Schedule M of the Food and Drug Regulations (C.R.C., c. 870), Sections B.01.001, B.01.002A and D.01.001, Government of Canada, Health Canada [21]. ^5^ Missing data indicates products which were evaluated, but values were missing for at least one of the tested nutrients that were required to determine whether a product would or would not meet the CAI criteria. However, in cases where values were missing for at least one of the tested nutrients but it did not affect the product’s evaluation (e.g., a “nutrient to encourage” had a missing value but the product already did not meet the “nutrients required to limit”), it was still possible to classify these products as or ‘restricted’ from marketing to children. Therefore, such products were not considered as having missing data. ^6^ Percentage of total products (i.e., out of *n* = 15,231 products). ^7^ Percentage of total products in that food category. ^8^ Percentage of products from CAI participating companies in that food category. ^9^ Percentage of products from non-participating companies in that food category.

**Table 4:** Proportion of packaged food and beverage products ^1^ with child-directed packaging ^2^ offered by CAI participating and non-participating companies ^3^ that would be allowed to be advertised to children according to the CAI’s Uniform Nutrition Criteria ^4^, presented overall and by food category ^5^.

Schedule M Category ^5^			Packaged Foods ^1^ from CAI Participating Companies ^3^								Packaged Foods ^1^ from Non-Participating Companies							
	Total Products Analyzed		Total Products Analyzed		Total Allowed ^4^		Total Restricted ^4^		No CAI Criteria ^4^		Total Products Analyzed		Total Allowed ^4^		Total Restricted ^4^		No CAI Criteria ^4^	
	*n*	% ^6^	*n*	% ^7^	*n*	% ^8^	*n*	% ^8^	*n*	% ^8^	*n*	% ^7^	*n*	% ^9^	*n*	% ^9^	*n*	% ^9^
TOTAL	747	100.0	249	33.3	76	30.5	173	69.5	0	0.0	498	66.7	99	19.9	383	76.9	16	3.2
1. Bakery Products	173	23.2	65	37.6	17	26.2	48	73.8	0	0.0	108	62.4	29	26.9	79	73.1	0	0.0
2. Beverages (soft drinks, iced teas, coffees)	11	1.5	4	36.4	0	0.0	4	100.0	0	0.0	7	63.6	0	0.0	7	100.0	0	0.0
3. Cereals, other grain products	51	6.8	33	64.7	23	69.7	10	30.3	0	0.0	18	35.3	2	11.1	16	88.9	0	0.0
4. Dairy products, substitutes	74	9.9	38	51.4	24	63.2	14	36.8	0	0.0	36	48.6	21	58.3	15	41.7	0	0.0
5. Desserts (Dairy and non-Dairy)	144	19.3	38	26.4	2	5.3	36	94.7	0	0.0	106	73.6	2	1.9	104	98.1	0	0.0
6. Dessert toppings, fillings	7	0.9	1	14.3	0	0.0	1	100.0	0	0.0	6	85.7	0	0.0	6	100.0	0	0.0
7. Eggs, egg substitutes	0	0.0	N/A		N/A		N/A		N/A		N/A		N/A		N/A		N/A	
8. Fats, oils	0	0.0	N/A		N/A		N/A		N/A		N/A		N/A		N/A		N/A	
9. Marine, fresh water animals	2	0.3	0	0.0	N/A		N/A		N/A		2	100.0	0	0.0	2	100.0	0	0.0
10. Fruit, fruit juices	58	7.8	30	51.7	5	16.7	25	83.3	0	0.0	28	48.3	13	46.4	15	53.6	0	0.0
11. Legumes	0	0.0	N/A		N/A		N/A		N/A		N/A		N/A		N/A		N/A	
12. Meat, poultry, their products, substitutes	4	0.5	0	0.0	N/A		N/A		N/A		4	100.0	1	25.0	3	75.0	0	0.0
13. Miscellaneous	14	1.9	1	7.1	0	0.0	1	100.0	0	0.0	13	92.9	0	0.0	7	53.8	6	46.2
14. Combination Dishes	69	9.2	6	8.7	1	16.7	5	83.3	0	0.0	63	91.3	19	30.2	44	69.8	0	0.0
15. Nuts, seeds	9	1.2	2	22.2	0	0.0	2	100.0	0	0.0	7	77.8	4	57.1	3	42.9	0	0.0
16. Potatoes, sweet potatoes, yams	4	0.5	0	0.0	N/A		N/A		N/A		4	100.0	2	50.0	2	50.0	0	0.0
17. Salads	0	0.0	N/A		N/A		N/A		N/A		N/A		N/A		N/A		N/A	
18. Sauces, dips, gravies, condiments	0	0.0	N/A		N/A		N/A		N/A		N/A		N/A		N/A		N/A	
19. Snacks (Chips, pretzels, meat or nut snacks)	54	7.2	10	18.5	4	40.0	6	60.0	0	0.0	44	81.5	6	13.6	38	86.4	0	0.0
20. Soups	1	0.1	0	0.0	N/A		N/A		N/A		1	100.0	0	0.0	1	100.0	0	0.0
21. Sugars, sweets	72	9.6	21	29.2	0	0.0	21	100.0	0	0.0	51	70.8	0	0.0	41	80.4	10	19.6
22. Vegetables	0	0.0	N/A		N/A		N/A		N/A		N/A		N/A		N/A		N/A	

^1^ Packaged food products are from the Food Label Information Program (FLIP) 2013 database, as described in [22]. ^2^ Products with child-directed packaging were identified using criteria based on previous publications [23]. ^3^ Companies Participating in the CAI, as Defined by the CAI Core Principles, October 2017 [16]. ^4^ Canadian Children’s Food and Beverage Advertising Initiative’s (CAI) Uniform Nutrition Criteria are defined in: Advertising Standards Canada. *Canadian Children’s Food and Beverage Advertising Initiative*; Uniform Nutrition Criteria White Paper; September 2014 [17]. ^5^ Food categories are defined in Schedule M of the Food and Drug Regulations (C.R.C., c. 870), Sections B.01.001, B.01.002A and D.01.001, Government of Canada, Health Canada [21]. ^6^ Percentage of total products (i.e., out of *n* = 747 products). ^7^ Percentage of total products in that food category. ^8^ Percentage of products from CAI participating companies in that food category. ^9^ Percentage of products from non-participating companies in that food category.

## Appendix A

**Table A1:** Evaluation of Canadian packaged food and beverage products ^1^ according to the Canadian Children’s Food and Beverage Advertising Initiative’s (CAI) Uniform Nutrition Criteria ^2^, presented by food subcategory ^3^.

Schedule M Category/Subcategory ^3^					CAI Uniform Nutrition Criteria ^2^													
					Allowed						Restricted							
	Total Products		Missing Data ^4^		Meets CAI Criteria ^2^		Automatically Allowed ^2^		Total Allowed		Does Not meet CAI Criteria ^2^		Automatically Restricted ^2^		Total Restricted		No Criteria ^2^	
	*n*	% ^5^	*n*	% ^6^	*n*	% ^6^	*n*	% ^6^	*n*	% ^6^	*n*	% ^6^	*n*	% ^6^	*n*	% ^6^	*n*	% ^6^
TOTAL	152,314	100.0	7	0.0	3278	21.5	572	3.8	3850	25.3	7606	49.9	1105	7.3	8711	57.2	2663	17.5
1. Bakery Products	2084	13.7	2	0.1	470	22.6	0	0.0	470	22.6	1612	77.4	0	0.0	1612	77.4	0	0.0
1. Bread (excl. sweet quick-type rolls)	231	1.5	0	0.0	65	28.1	0	0.0	65	28.1	166	71.9	0	0.0	166	71.9	0	0.0
2. Bagels, scones, rolls, buns, tortillas, etc.	288	1.9	0	0.0	57	19.8	0	0.0	57	19.8	231	80.2	0	0.0	231	80.2	0	0.0
3. Brownies	28	0.2	0	0.0	3	10.7	0	0.0	3	10.7	25	89.3	0	0.0	25	89.3	0	0.0
4. Heavy weight cake (e.g., cheese cake, cake with fruits and nuts)	44	0.3	0	0.0	0	0.0	0	0.0	0	0.0	44	100.0	0	0.0	44	100.0	0	0.0
5. Medium weight cake (e.g., Boston cream pie, cream puffs, cupcakes)	80	0.5	0	0.0	0	0.0	0	0.0	0	0.0	80	100.0	0	0.0	80	100.0	0	0.0
6. Light weight cake (e.g., angel food, chiffon, without icing or filling)	6	0.0	0	0.0	0	0.0	0	0.0	0	0.0	6	100.0	0	0.0	6	100.0	0	0.0
7. Coffee cakes, doughnuts, danishes, muffins, sweet quick-type rolls, etc.	123	0.8	0	0.0	8	6.5	0	0.0	8	6.5	115	93.5	0	0.0	115	93.5	0	0.0
8. Cookies, with or without coating/filling, graham wafers	391	2.6	0	0.0	70	17.9	0	0.0	70	17.9	321	82.1	0	0.0	321	82.1	0	0.0
9. Crackers, hard bread sticks, melba toast	277	1.8	0	0.0	125	45.1	0	0.0	125	45.1	152	54.9	0	0.0	152	54.9	0	0.0
10. Dry breaks, matzo, rusks	67	0.4	0	0.0	33	49.3	0	0.0	33	49.3	34	50.7	0	0.0	34	50.7	0	0.0
11. Flaky type pastries, with or without filling/icing	15	0.1	0	0.0	2	13.3	0	0.0	2	13.3	13	86.7	0	0.0	13	86.7	0	0.0
12. Toaster pastries	11	0.1	0	0.0	0	0.0	0	0.0	0	0.0	11	100.0	0	0.0	11	100.0	0	0.0
13. Ice cream cones	27	0.2	0	0.0	6	22.2	0	0.0	6	22.2	21	77.8	0	0.0	21	77.8	0	0.0
14. Croutons	37	0.2	1	2.7	4	10.8	0	0.0	4	10.8	32	86.5	0	0.0	32	86.5	0	0.0
15. French toast, pancakes, waffles	59	0.4	0	0.0	13	22.0	0	0.0	13	22.0	46	78.0	0	0.0	46	78.0	0	0.0
16. Grain-based bars, with filling or coating	105	0.7	0	0.0	22	21.0	0	0.0	22	21.0	83	79.0	0	0.0	83	79.0	0	0.0
17. Grain-based bars, without filling or coating	100	0.7	0	0.0	41	41.0	0	0.0	41	41.0	59	59.0	0	0.0	59	59.0	0	0.0
18. Rice cakes and corn cakes	51	0.3	0	0.0	18	35.3	0	0.0	18	35.3	33	64.7	0	0.0	33	64.7	0	0.0
19. Pies, tarts, cobblers, other pastries	101	0.7	0	0.0	0	0.0	0	0.0	0	0.0	101	100.0	0	0.0	101	100.0	0	0.0
20. Pie crust	18	0.1	0	0.0	0	0.0	0	0.0	0	0.0	18	100.0	0	0.0	18	100.0	0	0.0
21. Pizza Crust	11	0.1	0	0.0	0	0.0	0	0.0	0	0.0	11	100.0	0	0.0	11	100.0	0	0.0
22. Taco shell, hard	14	0.1	1	7.1	3	21.4	0	0.0	3	21.4	10	71.4	0	0.0	10	71.4	0	0.0
2. Beverages (soft drinks, iced teas, coffees)	482	3.2	0	0.0	0	0.0	0	0.0	0	0.0	0	0.0	482	100.0	482	100.0	0	0.0
23. Carbonated and non-carbonated beverages, wine coolers	264	1.7	0	0.0	0	0.0	0	0.0	0	0.0	0	0.0	264	100.0	264	100.0	0	0.0
24. Sports drinks and water	125	0.8	0	0.0	0	0.0	0	0.0	0	0.0	0	0.0	125	100.0	125	100.0	0	0.0
25. Coffee, espresso, flavored/unflavored	29	0.2	0	0.0	0	0.0	0	0.0	0	0.0	0	0.0	29	100.0	29	100.0	0	0.0
26. Tea and herbal tea, flavored/unflavored	37	0.2	0	0.0	0	0.0	0	0.0	0	0.0	0	0.0	37	100.0	37	100.0	0	0.0
27. Cocoa and chocolate beverages (hot)	27	0.2	0	0.0	0	0.0	0	0.0	0	0.0	0	0.0	27	100.0	27	100.0	0	0.0
3. Cereals, other grain products	988	6.5	1	0.1	332	33.6	0	0.0	332	33.6	592	59.9	0	0.0	592	59.9	63	6.4
28. Hot breakfast cereals (oatmeal, cream of wheat)	105	0.7	1	1.0	92	87.6	0	0.0	92	87.6	12	11.4	0	0.0	12	11.4	0	0.0
29. Ready-to-eat breakfast cereals, puffed and uncoated	2	0.0	0	0.0	0	0.0	0	0.0	0	0.0	2	100.0	0	0.0	2	100.0	0	0.0
30. Ready-to-eat breakfast cereals, puffed and coated, flaked, extruded, without fruit/nuts, high fiber cereal	77	0.5	0	0.0	43	55.8	0	0.0	43	55.8	34	44.2	0	0.0	34	44.2	0	0.0
31. Ready-to-eat breakfast cereals, with fruit/nuts, granola, biscuit type cereals	171	1.1	0	0.0	63	36.8	0	0.0	63	36.8	108	63.2	0	0.0	108	63.2	0	0.0
32. Bran and wheat germ	5	0.0	0	0.0	5	100.0	0	0.0	5	100.0	0	0.0	0	0.0	0	0.0	0	0.0
33. Flours (incl. cornmeal)	50	0.3	0	0.0	0	0.0	0	0.0	0	0.0	0	0.0	0	0.0	0	0.0	50	100.0
34. Grains (e.g., rice, barley, etc.)	110	0.7	0	0.0	51	46.4	0	0.0	51	46.4	59	53.6	0	0.0	59	53.6	0	0.0
35. Pastas without sauce	437	2.9	0	0.0	75	17.2	0	0.0	75	17.2	362	82.8	0	0.0	362	82.8	0	0.0
37. Starch (e.g., cornstarch, potato starch, tapioca starch, etc.)	13	0.1	0	0.0	0	0.0	0	0.0	0	0.0	0	0.0	0	0.0	0	0.0	13	100.0
38. Stuffing	18	0.1	0	0.0	3	16.7	0	0.0	3	16.7	15	83.3	0	0.0	15	83.3	0	0.0
4. Dairy products, substitutes	1240	8.1	1	0.1	645	52.0	79	6.4	724	58.4	515	41.5	0	0.0	515	41.5	0	0.0
39. Cheese (incl. cream cheese and cheese spread), except those listed as separate item	453	3.0	1	0.2	324	71.5	0	0.0	324	71.5	128	28.3	0	0.0	128	28.3	0	0.0
40. Cottage cheese	27	0.2	0	0.0	21	77.8	0	0.0	21	77.8	6	22.2	0	0.0	6	22.2	0	0.0
41. Cheese, as an ingredient (e.g., dry cottage cheese, ricotta, etc.)	17	0.1	0	0.0	3	17.6	0	0.0	3	17.6	14	82.4	0	0.0	14	82.4	0	0.0
42. Hard cheese, grated (e.g., parmesan, romano)	30	0.2	0	0.0	27	90.0	0	0.0	27	90.0	3	10.0	0	0.0	3	10.0	0	0.0
43. Quark, fresh cheese, fresh dairy desserts	99	0.7	0	0.0	2	2.0	0	0.0	2	2.0	97	98.0	0	0.0	97	98.0	0	0.0
44. Cream and cream substitute	50	0.3	0	0.0	0	0.0	0	0.0	0	0.0	50	100.0	0	0.0	50	100.0	0	0.0
45. Cream and cream substitute, powder	13	0.1	0	0.0	0	0.0	0	0.0	0	0.0	13	100.0	0	0.0	13	100.0	0	0.0
46. Cream and cream substitute, aerosol or whipped	22	0.1	0	0.0	0	0.0	0	0.0	0	0.0	22	100.0	0	0.0	22	100.0	0	0.0
48. Milk, evaporated or condensed	18	0.1	0	0.0	0	0.0	0	0.0	0	0.0	18	100.0	0	0.0	18	100.0	0	0.0
49. Plant-based beverages, milk, buttermilk, milk-based drinks (e.g., chocolate milk)	246	1.6	0	0.0	118	48.0	79	32.1	197	80.1	49	19.9	0	0.0	49	19.9	0	0.0
50. Shakes and shake substitutes (e.g., dairy shake mix)	11	0.1	0	0.0	3	27.3	0	0.0	3	27.3	8	72.7	0	0.0	8	72.7	0	0.0
51. Sour cream	18	0.1	0	0.0	0	0.0	0	0.0	0	0.0	18	100.0	0	0.0	18	100.0	0	0.0
52. Yogurt	236	1.6	0	0.0	147	62.3	0	0.0	147	62.3	89	37.7	0	0.0	89	37.7	0	0.0
5. Desserts (Dairy and non-Dairy)	827	5.4	0	0.0	59	7.1	0	0.0	59	7.1	767	92.7	0	0.0	767	92.7	1	0.1
53. Ice cream, ice milk, frozen yogurt and sherbet	389	2.6	0	0.0	46	11.8	0	0.0	46	11.8	343	88.2	0	0.0	343	88.2	0	0.0
54. Dairy desserts, frozen (e.g., cakes, bars, sandwiches, cones)	186	1.2	0	0.0	3	1.6	0	0.0	3	1.6	183	98.4	0	0.0	183	98.4	0	0.0
55. Non-dairy desserts, frozen (e.g., flavored/sweetened ice or pops, frozen fruit juices in bars or cups)	46	0.3	0	0.0	5	10.9	0	0.0	5	10.9	41	89.1	0	0.0	41	89.1	0	0.0
56. Sundaes	9	0.1	0	0.0	0	0.0	0	0.0	0	0.0	9	100.0	0	0.0	9	100.0	0	0.0
57. Custard, gelatin, pudding	197	1.3	0	0.0	5	2.5	0	0.0	5	2.5	191	97.0	0	0.0	191	97.0	1	0.5
6. Dessert toppings, fillings	116	0.8	0	0.0	0	0.0	0	0.0	0	0.0	0	0.0	116	100.0	116	100.0	0	0.0
58. Dessert toppings (e.g., maple butter, marshmallow cream)	30	0.2	0	0.0	0	0.0	0	0.0	0	0.0	0	0.0	30	100.0	30	100.0	0	0.0
59. Cake frostings and icings	58	0.4	0	0.0	0	0.0	0	0.0	0	0.0	0	0.0	58	100.0	58	100.0	0	0.0
60. Pie fillings	28	0.2	0	0.0	0	0.0	0	0.0	0	0.0	0	0.0	28	100.0	28	100.0	0	0.0
7. Eggs, egg substitutes	56	0.4	0	0.0	42	75.0	0	0.0	42	75.0	14	25.0	0	0.0	14	25.0	0	0.0
61. Egg mixtures (e.g., egg foo young, scrambled eggs, omelets)	7	0.1	0	0.0	7	100.0	0	0.0	7	100.0	0	0.0	0	0.0	0	0.0	0	0.0
62. Eggs	49	0.3	0	0.0	35	71.4	0	0.0	35	71.4	14	28.6	0	0.0	14	28.6	0	0.0
8. Fats, oils	535	3.5	0	0.0	0	0.0	0	0.0	0	0.0	0	0.0	0	0.0	0	0.0	535	100.0
64. Butter, margarine, shortening, lard	91	0.6	0	0.0	0	0.0	0	0.0	0	0.0	0	0.0	0	0.0	0	0.0	91	100.0
65. Vegetable oil	136	0.9	0	0.0	0	0.0	0	0.0	0	0.0	0	0.0	0	0.0	0	0.0	136	100.0
67. Dressings for salad	252	1.7	0	0.0	0	0.0	0	0.0	0	0.0	0	0.0	0	0.0	0	0.0	252	100.0
68. Mayonnaise, sandwich spread and mayonnaise-type dressing	40	0.3	0	0.0	0	0.0	0	0.0	0	0.0	0	0.0	0	0.0	0	0.0	40	100.0
69. Oil, spray type	16	0.1	0	0.0	0	0.0	0	0.0	0	0.0	0	0.0	0	0.0	0	0.0	16	100.0
9. Marine, fresh water animals	440	2.9	0	0.0	81	18.4	0	0.0	81	18.4	359	81.6	0	0.0	359	81.6	0	0.0
70. Canned anchovies, anchovy paste, caviar	6	0.0	0	0.0	0	0.0	0	0.0	0	0.0	6	100.0	0	0.0	6	100.0	0	0.0
71. Marine and fresh water animals with sauce (e.g., shrimp with lobster sauce)	40	0.3	0	0.0	5	12.5	0	0.0	5	12.5	35	87.5	0	0.0	35	87.5	0	0.0
72. Marine and fresh water animals without sauce (e.g., plain or fried fish/shellfish, fish cakes) with or without breading/batter	209	1.4	0	0.0	24	11.5	0	0.0	24	11.5	185	88.5	0	0.0	185	88.5	0	0.0
73. Marine and fresh water animals, canned	129	0.9	0	0.0	50	38.8	0	0.0	50	38.8	79	61.2	0	0.0	79	61.2	0	0.0
74. Marine and fresh water animals, smoked or pickled, or spreads	56	0.4	0	0.0	2	3.6	0	0.0	2	3.6	54	96.4	0	0.0	54	96.4	0	0.0
10. Fruit, fruit juices	1089	7.2	0	0.0	489	44.9	58	5.3	547	50.2	528	48.5	0	0.0	528	48.5	14	1.3
75. Fruit, fresh, canned or frozen, except those listed as separate item	278	1.8	0	0.0	120	43.2	48	17.3	168	60.4	110	39.6	0	0.0	110	39.6	0	0.0
76. Candied or pickled fruit	5	0.0	0	0.0	0	0.0	0	0.0	0	0.0	5	100.0	0	0.0	5	100.0	0	0.0
77. Dried fruit (e.g., raisins, dates, figs)	141	0.9	0	0.0	21	14.9	0	0.0	21	14.9	120	85.1	0	0.0	120	85.1	0	0.0
78. Fruit for garnish or flavor (e.g., maraschino cherries)	5	0.0	0	0.0	0	0.0	0	0.0	0	0.0	5	100.0	0	0.0	5	100.0	0	0.0
79. Fruit relishes	14	0.1	0	0.0	0	0.0	0	0.0	0	0.0	0	0.0	0	0.0	0	0.0	14	100.0
81. Cranberries, lemons, limes, used as ingredients	3	0.0	0	0.0	0	0.0	3	100.0	3	100.0	0	0.0	0	0.0	0	0.0	0	0.0
83. Juices, nectars and fruit drinks for use as substitutes for fruit juices	636	4.2	0	0.0	348	54.7	0	0.0	348	54.7	288	45.3	0	0.0	288	45.3	0	0.0
84. Juices, used as ingredients (e.g., lemon or lime juice)	7	0.1	0	0.0	0	0.0	7	100.0	7	100.0	0	0.0	0	0.0	0	0.0	0	0.0
11. Legumes	180	1.2	0	0.0	89	49.4	0	0.0	89	49.4	91	50.6	0	0.0	91	50.6	0	0.0
85. Bean curd (tofu) and tempeh	16	0.1	0	0.0	3	18.8	0	0.0	3	18.8	13	81.3	0	0.0	13	81.3	0	0.0
86. Beans, peas, lentils (e.g., chickpeas, kidney beans, soybeans)	164	1.1	0	0.0	86	52.4	0	0.0	86	52.4	78	47.6	0	0.0	78	47.6	0	0.0
12. Meat, poultry, their products, substitutes	895	5.9	0	0.0	77	8.6	0	0.0	77	8.6	818	91.4	0	0.0	818	91.4	0	0.0
87. Pork rinds and bacon	38	0.3	0	0.0	0	0.0	0	0.0	0	0.0	38	100.0	0	0.0	38	100.0	0	0.0
88. Beef, pork and poultry breakfast strips	11	0.1	0	0.0	2	18.2	0	0.0	2	18.2	9	81.8	0	0.0	9	81.8	0	0.0
89. Dried meat and poultry (e.g., jerky, Parma ham) and dry sausage products (e.g., salami)	77	0.5	0	0.0	0	0.0	0	0.0	0	0.0	77	100.0	0	0.0	77	100.0	0	0.0
90. Luncheon meats (e.g., bologna, mortadella), pâté, sandwich spread, taco fillings, meat pie fillings	101	0.7	0	0.0	7	6.9	0	0.0	7	6.9	94	93.1	0	0.0	94	93.1	0	0.0
91. Sausage products (e.g., wieners breakfast sausage, pepperoni, frankfurters)	172	1.1	0	0.0	6	3.5	0	0.0	6	3.5	166	96.5	0	0.0	166	96.5	0	0.0
92. Cuts or meat and poultry without sauce, and ready-to-cook cuts, with or without breading/batter, including marinated, tenderized, injected cuts	123	0.8	0	0.0	14	11.4	0	0.0	14	11.4	109	88.6	0	0.0	109	88.6	0	0.0
93. Patties, cutlets, choppettes, steakettes, meatballs, sausage meat, ground meat, with or without breading/batter	191	1.3	0	0.0	30	15.7	0	0.0	30	15.7	161	84.3	0	0.0	161	84.3	0	0.0
94. Cured meat products (e.g., cured ham, back bacon, corned beef, smoked meat etc.)	86	0.6	0	0.0	2	2.3	0	0.0	2	2.3	84	97.7	0	0.0	84	97.7	0	0.0
95. Canned meat and poultry	23	0.2	0	0.0	1	4.3	0	0.0	1	4.3	22	95.7	0	0.0	22	95.7	0	0.0
96. Meat and poultry with sauce (e.g., meat in barbeque sauce, turkey with gravy), excluding combination dishes	73	0.5	0	0.0	15	20.6	0	0.0	15	20.6	58	79.5	0	0.0	58	79.5	0	0.0
13. Miscellaneous	450	3.0	0	0.0	18	4.0	0	0.0	18	4.0	193	42.9	11	2.4	204	45.3	228	50.7
97. Baking powder, baking soda, pectin	5	0.0	0	0.0	0	0.0	0	0.0	0	0.0	0	0.0	0	0.0	0	0.0	5	100.0
98. Baking decorations (e.g., colored sugars, sprinkles)	6	0.0	0	0.0	0	0.0	0	0.0	0	0.0	0	0.0	6	100.0	6	100.0	0	0.0
99. Bread crumbs and batter mixes	211	1.4	0	0.0	18	8.5	0	0.0	18	8.5	193	91.5	0	0.0	193	91.5	0	0.0
101. Cocoa powder	4	0.0	0	0.0	0	0.0	0	0.0	0	0.0	0	0.0	0	0.0	0	0.0	4	100.0
102. Non-alcoholic drink mixers (e.g., piña colada, daiquiri)	5	0.0	0	0.0	0	0.0	0	0.0	0	0.0	0	0.0	5	100.0	5	100.0	0	0.0
104. Salad and potato toppers (e.g., salad crunchies, crispins, substitutes for bacon bits)	28	0.2	0	0.0	0	0.0	0	0.0	0	0.0	0	0.0	0	0.0	0	0.0	28	100.0
105. Salt and salt substitutes, seasoned salts (e.g., garlic salt)	17	0.1	0	0.0	0	0.0	0	0.0	0	0.0	0	0.0	0	0.0	0	0.0	17	100.0
106. Spices and herbs	170	1.1	0	0.0	0	0.0	0	0.0	0	0.0	0	0.0	0	0.0	0	0.0	170	100.0
14. Combination Dishes	1357	8.9	3	0.2	473	34.9	0	0.0	473	34.9	881	64.9	0	0.0	881	64.9	0	0.0
107. Measurable with a cup (e.g., casserole, macaroni and cheese, stir fry, chili, ravioli in sauce, poutine)	671	4.4	0	0.0	197	29.4	0	0.0	197	29.4	474	70.6	0	0.0	474	70.6	0	0.0
108. Not measurable with a cup (e.g., burritos, egg rolls, pizza, quiche, stuffed vegetables, shish kabobs, meat and poultry lunch packages)	522	3.4	1	0.2	153	29.3	0	0.0	153	29.3	368	70.5	0	0.0	368	70.5	0	0.0
109. Meat pie and tourtière	164	1.1	2	1.2	123	75.0	0	0.0	123	75.0	39	23.8	0	0.0	39	23.8	0	0.0
15. Nuts, seeds	220	1.4	0	0.0	126	57.3	0	0.0	126	57.3	87	39.6	0	0.0	87	39.6	7	3.2
110. Nuts and seeds, not for use as snacks, whole, chopped, sliced, slivered, ground	130	0.9	0	0.0	101	77.7	0	0.0	101	77.7	29	22.3	0	0.0	29	22.3	0	0.0
111. Butters, pastes, creams (excl. peanut butter)	33	0.2	0	0.0	25	75.8	0	0.0	25	75.8	8	24.2	0	0.0	8	24.2	0	0.0
112. Peanut butter	50	0.3	0	0.0	0	0.0	0	0.0	0	0.0	50	100.0	0	0.0	50	100.0	0	0.0
113. Flours (e.g., coconut flour)	7	0.1	0	0.0	0	0.0	0	0.0	0	0.0	0	0.0	0	0.0	0	0.0	7	100.0
16. Potatoes, sweet potatoes, yams	140	0.9	0	0.0	74	52.9	0	0.0	74	52.9	66	47.1	0	0.0	66	47.1	0	0.0
114. French fried, hash browns, skins and pancakes	69	0.5	0	0.0	57	82.6	0	0.0	57	82.6	12	17.4	0	0.0	12	17.4	0	0.0
115. Mashed, candied, stuffed or with sauce	56	0.4	0	0.0	4	7.1	0	0.0	4	7.1	52	92.9	0	0.0	52	92.9	0	0.0
116. Plain, fresh, canned or frozen	15	0.1	0	0.0	13	86.7	0	0.0	13	86.7	2	13.3	0	0.0	2	13.3	0	0.0
17. Salads	70	0.5	0	0.0	48	68.6	0	0.0	48	68.6	22	31.4	0	0.0	22	31.4	0	0.0
117. Salads (e.g., Egg, fish, shellfish, bean, fruit, vegetable, meat), except those as separate item	46	0.3	0	0.0	34	73.9	0	0.0	34	73.9	12	26.1	0	0.0	12	26.1	0	0.0
119. Pasta or potato salad	24	0.2	0	0.0	14	58.3	0	0.0	14	58.3	10	41.7	0	0.0	10	41.7	0	0.0
18. Sauces, dips, gravies, condiments	1229	8.1	0	0.0	0	0.0	0	0.0	0	0.0	0	0.0	0	0.0	0	0.0	1229	100.0
120. Sauces for dipping (e.g., barbeque, hollandaise, tartar, mustard, sweet and sour)	104	0.7	0	0.0	0	0.0	0	0.0	0	0.0	0	0.0	0	0.0	0	0.0	104	100.0
121. Dips (legume or dairy-based)	140	0.9	0	0.0	0	0.0	0	0.0	0	0.0	0	0.0	0	0.0	0	0.0	140	100.0
122. Major main entrée sauce (e.g., spaghetti sauce)	225	1.5	0	0.0	0	0.0	0	0.0	0	0.0	0	0.0	0	0.0	0	0.0	225	100.0
123. Minor main entrée sauce (e.g., pizza sauce, pesto sauce, salsa, etc.)	287	1.9	0	0.0	0	0.0	0	0.0	0	0.0	0	0.0	0	0.0	0	0.0	287	100.0
124. Major condiments (e.g., ketchup, steak sauce, vinegar, marinades)	375	2.5	0	0.0	0	0.0	0	0.0	0	0.0	0	0.0	0	0.0	0	0.0	375	100.0
125. Minor condiments (e.g., horseradish, hot sauce, mustard, Worcestershire sauce)	98	0.6	0	0.0	0	0.0	0	0.0	0	0.0	0	0.0	0	0.0	0	0.0	98	100.0
19. Snacks (Chips, pretzels, meat or nut snacks)	794	5.2	0	0.0	167	21.0	0	0.0	167	21.0	627	79.0	0	0.0	627	79.0	0	0.0
126. Chips, pretzels, popcorn, extruded snacks, grain-based snack mixes and fruit-based snacks (e.g., fruit chips)	558	3.7	0	0.0	87	15.6	0	0.0	87	15.6	471	84.4	0	0.0	471	84.4	0	0.0
127. Nuts or seeds for use as snacks	223	1.5	0	0.0	80	35.9	0	0.0	80	35.9	143	64.1	0	0.0	143	64.1	0	0.0
128. Meat or poultry snack food sticks	13	0.1	0	0.0	0	0.0	0	0.0	0	0.0	13	100.0	0	0.0	13	100.0	0	0.0
20. Soups	456	3.0	0	0.0	69	15.1	0	0.0	69	15.1	387	84.9	0	0.0	387	84.9	0	0.0
129. All varieties	456	3.0	0	0.0	69	15.1	0	0.0	69	15.1	387	84.9	0	0.0	387	84.9	0	0.0
21. Sugars, sweets	749	4.9	0	0.0	0	0.0	0	0.0	0	0.0	0	0.0	496	66.2	496	66.2	253	33.8
130. Candies (incl. chocolate bars and chocolate products), except those as separate item	377	2.5	0	0.0	0	0.0	0	0.0	0	0.0	0	0.0	377	100.0	377	100.0	0	0.0
131. Hard candies, except those as separate item	8	0.1	0	0.0	0	0.0	0	0.0	0	0.0	0	0.0	8	100.0	8	100.0	0	0.0
132. Baking candies, such as chocolate chips	30	0.2	0	0.0	0	0.0	0	0.0	0	0.0	0	0.0	30	100.0	30	100.0	0	0.0
133. Breath mints	6	0.0	0	0.0	0	0.0	0	0.0	0	0.0	0	0.0	6	100.0	6	100.0	0	0.0
135. Confectioner’s or icing sugar	1	0.0	0	0.0	0	0.0	0	0.0	0	0.0	0	0.0	0	0.0	0	0.0	1	100.0
136. Bread spreads, except those as separate item, honey, molasses	50	0.3	0	0.0	0	0.0	0	0.0	0	0.0	0	0.0	0	0.0	0	0.0	50	100.0
137. Jams, jellies, marmalades, fruit butters, spreads	193	1.3	0	0.0	0	0.0	0	0.0	0	0.0	0	0.0	0	0.0	0	0.0	193	100.0
138. Marshmallows	13	0.1	0	0.0	0	0.0	0	0.0	0	0.0	0	0.0	13	100.0	13	100.0	0	0.0
139. Sugars, except those as separate item	6	0.0	0	0.0	0	0.0	0	0.0	0	0.0	0	0.0	0	0.0	0	0.0	6	100.0
140. Sugar substitute	3	0.0	0	0.0	0	0.0	0	0.0	0	0.0	0	0.0	0	0.0	0	0.0	3	100.0
141. Syrups (incl. chocolate, maple and corn syrup)	62	0.4	0	0.0	0	0.0	0	0.0	0	0.0	0	0.0	62	100.0	62	100.0	0	0.0
22. Vegetables	834	5.5	0	0.0	19	2.3	435	52.2	454	54.4	47	5.6	0	0.0	47	5.6	333	39.9
142. Vegetables without sauce (incl. cream style corn, stewed tomatoes), except vegetables without sauce listed as separate item	418	2.7	0	0.0	0	0.0	418	100.0	418	100.0	0	0.0	0	0.0	0	0.0	0	0.0
143. Vegetables with sauce	17	0.1	0	0.0	14	82.4	0	0.0	14	82.4	3	17.6	0	0.0	3	17.6	0	0.0
144. Vegetables for use as garnish/flavoring, fresh, canned or frozen, but not dried (e.g., parsley or garlic)	24	0.2	0	0.0	0	0.0	0	0.0	0	0.0	0	0.0	0	0.0	0	0.0	24	100.0
145. Chili pepper and green onion	42	0.3	0	0.0	0	0.0	0	0.0	0	0.0	0	0.0	0	0.0	0	0.0	42	100.0
146. Seaweed	6	0.0	0	0.0	1	16.7	0	0.0	1	16.7	5	83.3	0	0.0	5	83.3	0	0.0
147. Lettuce and spouts	17	0.1	0	0.0	0	0.0	17	100.0	17	100.0	0	0.0	0	0.0	0	0.0	0	0.0
148. Vegetable juice and vegetable drink	43	0.3	0	0.0	4	9.3	0	0.0	4	9.3	39	90.7	0	0.0	39	90.7	0	0.0
149. Olives	83	0.5	0	0.0	0	0.0	0	0.0	0	0.0	0	0.0	0	0.0	0	0.0	83	100.0
150. Pickles	112	0.7	0	0.0	0	0.0	0	0.0	0	0.0	0	0.0	0	0.0	0	0.0	112	100.0
151. Relish	24	0.2	0	0.0	0	0.0	0	0.0	0	0.0	0	0.0	0	0.0	0	0.0	24	100.0
152. Vegetable pastes, (e.g., tomato paste)	12	0.1	0	0.0	0	0.0	0	0.0	0	0.0	0	0.0	0	0.0	0	0.0	12	100.0
153. Vegetable sauce or puree (e.g., tomato sauce or tomato puree)	36	0.2	0	0.0	0	0.0	0	0.0	0	0.0	0	0.0	0	0.0	0	0.0	36	100.0

^1^ Packaged food products are from the Food Label Information Program (FLIP) 2013, as described in [22]. ^2^ Canadian Children’s Food and Beverage Advertising Initiative’s (CAI) Uniform Nutrition Criteria are defined in: Advertising Standards Canada. *Canadian Children’s Food and Beverage Advertising Initiative*; Uniform Nutrition Criteria White Paper; September 2014 [17]. ^3^ Food categories are defined in Schedule M of the Food and Drug Regulations (C.R.C., c. 870), Sections B.01.001, B.01.002A and D.01.001, Government of Canada, Health Canada [21]. ^4^ Missing data indicates products which were evaluated, but values were missing for at least one of the tested nutrients that were required to determine whether a product would or would not meet the CAI criteria. However, in cases where values were missing for at least one of the tested nutrients but it did not affect the product’s evaluation (e.g., a “nutrient to encourage” had a missing value but the product already did not meet the “nutrients required to limit), it was still possible to classify these products as or ‘restricted’ from marketing to children, therefore such products were not considered as having missing data. ^5^ Percentage of total products (i.e., out of *n* = 15,231 products). ^6^ Percentage of total products in that food category/subcategory.

**Table A2:** Percentage of packaged food and beverage products ^1^ with child-directed packaging ^2^ that would be allowed to be advertised to children according to the CAI’s Uniform Nutrition Criteria ^3^, presented by food subcategory ^4^.

Schedule M Category/Subcategory ^4^	All Packaged Foods ^1^		Packaged Foods with Child-Directed Packaging ^2^							
	Total Products Analyzed		Total Products Analyzed		Total Allowed ^3^		Total Restricted ^3^		No CAI Criteria ^3^	
	*n*	% ^5^	*n*	% ^6^	*n*	% ^7^	*n*	% ^7^	*n*	% ^7^
TOTAL	15,231	100.0	747	4.9	175	23.4	556	74.4	16	2.1
1. Bakery Products	2084	13.7	173	8.3	46	26.6	127	73.4	0	0.0
1. Bread (excl. sweet quick-type rolls)	231	1.5	1	0.4	0	0.0	1	100.0	0	0.0
2. Bagels, scones, rolls, buns, tortillas, etc.	288	1.9	2	0.7	0	0.0	2	100.0	0	0.0
3. Brownies	28	0.2	2	7.1	0	0.0	2	100.0	0	0.0
4. Heavy weight cake (e.g., cheese cake, cake with fruits and nuts)	44	0.3	0	0.0	N/A		N/A		N/A	
5. Medium weight cake (e.g., Boston cream pie, cream puffs, cupcakes)	80	0.5	13	16.3	0	0.0	13	100.0	0	0.0
6. Light weight cake (e.g., angel food, chiffon, without icing or filling)	6	0.0	0	0.0	N/A		N/A		N/A	
7. Coffee cakes, doughnuts, danishes, muffins, sweet quick-type rolls, etc.	123	0.8	6	4.9	0	0.0	6	100.0	0	0.0
8. Cookies, with or without coating/filling, graham wafers	391	2.6	84	21.5	30	35.7	54	64.3	0	0.0
9. Crackers, hard bread sticks, melba toast	277	1.8	31	11.2	7	22.6	24	77.4	0	0.0
10. Dry breaks, matzo, rusks	67	0.4	0	0.0	N/A		N/A		N/A	
11. Flaky type pastries, with or without filling/icing	15	0.1	0	0.0	N/A		N/A		N/A	
12. Toaster pastries	11	0.1	11	100.0	0	0.0	11	100.0	0	0.0
13. Ice cream cones	27	0.2	1	3.7	0	0.0	1	100.0	0	0.0
14. Croutons	37	0.2	0	0.0	N/A		N/A		N/A	
15. French toast, pancakes, waffles	59	0.4	1	1.7	0	0.0	1	100.0	0	0.0
16. Grain-based bars, with filling or coating	105	0.7	9	8.6	2	22.2	7	77.8	0	0.0
17. Grain-based bars, without filling or coating	100	0.7	12	12.0	7	58.3	5	41.7	0	0.0
18. Rice cakes and corn cakes	51	0.3	0	0.0	N/A		N/A		N/A	
19. Pies, tarts, cobblers, other pastries	101	0.7	0	0.0	N/A		N/A		N/A	
20. Pie crust	18	0.1	0	0.0	N/A		N/A		N/A	
21. Pizza Crust	11	0.1	0	0.0	N/A		N/A		N/A	
22. Taco shell, hard	14	0.1	0	0.0	N/A		N/A		N/A	
2. Beverages (soft drinks, iced teas, coffees)	482	3.2	11	2.3	0	0.0	11	100.0	0	0.0
23. Carbonated and non-carbonated beverages, wine coolers	264	1.7	7	2.6	0	0.0	7	100.0	0	0.0
24. Sports drinks and water	125	0.8	1	0.8	0	0.0	1	100.0	0	0.0
25. Coffee, espresso, flavored/unflavored	29	0.2	0	0.0	N/A		N/A		N/A	
26. Tea and herbal tea, flavored/unflavored	37	0.2	0	0.0	N/A		N/A		N/A	
27. Cocoa and chocolate beverages (hot)	27	0.2	3	11.1	0	0.0	3	100.0	0	0.0
3. Cereals, other grain products	988	6.5	51	5.2	25	49.0	26	51.0	0	0.0
28. Hot breakfast cereals (oatmeal, cream of wheat)	105	0.7	3	2.8	3	100.0	0	0.0	0	0.0
29. Ready-to-eat breakfast cereals, puffed and uncoated	2	0.0	0	0.0	N/A		N/A		N/A	
30. Ready-to-eat breakfast cereals, puffed and coated, flaked, extruded, without fruit/nuts, high fiber cereal	77	0.5	33	42.7	13	39.4	20	60.6	0	0.0
31. Ready-to-eat breakfast cereals, with fruit/nuts, granola, biscuit type cereals	171	1.1	14	8.2	9	64.3	5	35.7	0	0.0
32. Bran and wheat germ	5	0.0	0	0.0	N/A		N/A		N/A	
33. Flours (incl. cornmeal)	50	0.3	0	0.0	N/A		N/A		N/A	
34. Grains (e.g., rice, barley, etc.)	110	0.7	0	0.0	N/A		N/A		N/A	
35. Pastas without sauce	437	2.9	1	0.2	0	0.0	1	100.0	0	0.0
37. Starch (e.g., cornstarch, potato starch, tapioca starch, etc.)	13	0.1	0	0.0	N/A		N/A		N/A	
38. Stuffing	18	0.1	0	0.0	N/A		N/A		N/A	
4. Dairy products, substitutes	1240	8.1	74	6.0	45	60.8	29	39.2	0	0.0
39. Cheese (incl. cream cheese and cheese spread), except those listed as separate item	453	3.0	16	3.5	13	81.3	3	18.8	0	0.0
40. Cottage cheese	27	0.2	0	0.0	N/A		N/A		N/A	
41. Cheese, as an ingredient (e.g., dry cottage cheese, ricotta, etc.)	17	0.1	0	0.0	N/A		N/A		N/A	
42. Hard cheese, grated (e.g., parmesan, romano)	30	0.2	0	0.0	N/A		N/A		N/A	
43. Quark, fresh cheese, fresh dairy desserts	99	0.7	8	8.1	1	12.5	7	87.5	0	0.0
44. Cream and cream substitute	50	0.3	0	0.0	N/A		N/A		N/A	
45. Cream and cream substitute, powder	13	0.1	0	0.0	N/A		N/A		N/A	
46. Cream and cream substitute, aerosol or whipped	22	0.1	0	0.0	N/A		N/A		N/A	
48. Milk, evaporated or condensed	18	0.1	0	0.0	N/A		N/A		N/A	
49. Plant-based beverages, milk, buttermilk, milk-based drinks (e.g., chocolate milk)	246	1.6	34	13.8	26	76.5	8	23.5	0	0.0
50. Shakes and shake substitutes (e.g., dairy shake mix)	11	0.1	5	45.5	0	0.0	5	100.0	0	0.0
51. Sour cream	18	0.1	0	0.0	N/A		N/A		N/A	
52. Yogurt	236	1.6	11	4.7	5	45.5	6	54.5	0	0.0
5. Desserts (Dairy and non-Dairy)	827	5.4	144	17.4	4	2.8	140	97.2	0	0.0
53. Ice cream, ice milk, frozen yogurt and sherbet	389	2.6	54	13.9	0	0.0	54	100.0	0	0.0
54. Dairy desserts, frozen (e.g., cakes, bars, sandwiches, cones)	186	1.2	43	23.0	1	2.3	42	97.7	0	0.0
55. Non-dairy desserts, frozen (e.g., flavored/sweetened ice or pops, frozen fruit juices in bars or cups)	46	0.3	24	52.2	2	8.3	22	91.7	0	0.0
56. Sundaes	9	0.1	3	33.3	0	0.0	3	100.0	0	0.0
57. Custard, gelatin, pudding	197	1.3	20	10.2	1	5.0	19	95.0	0	0.0
6. Dessert toppings, fillings	116	0.8	7	6.0	0	0.0	7	100.0	0	0.0
58. Dessert toppings (e.g., maple butter, marshmallow cream)	30	0.2	3	10.0	0	0.0	3	100.0	0	0.0
59. Cake frostings and icings	58	0.4	4	6.9	0	0.0	4	100.0	0	0.0
60. Pie fillings	28	0.2	0	0.0	N/A		N/A		N/A	
7. Eggs, egg substitutes	56	0.4	0	0.0	N/A		N/A		N/A	
61. Egg mixtures (e.g., egg foo young, scrambled eggs, omelets)	7	0.1	0	0.0	N/A		N/A		N/A	
62. Eggs	49	0.3	0	0.0	N/A		N/A		N/A	
8. Fats, oils	535	3.5	0	0.0	N/A		N/A		N/A	
64. Butter, margarine, shortening, lard	91	0.6	0	0.0	N/A		N/A		N/A	
65. Vegetable oil	136	0.9	0	0.0	N/A		N/A		N/A	
67. Dressings for salad	252	1.7	0	0.0	N/A		N/A		N/A	
68. Mayonnaise, sandwich spread and mayonnaise-type dressing	40	0.3	0	0.0	N/A		N/A		N/A	
69. Oil, spray type	16	0.1	0	0.0	N/A		N/A		N/A	
9. Marine, fresh water animals	440	2.9	2	0.5	0	0.0	2	100.0	0	0.0
70. Canned anchovies, anchovy paste, caviar	6	0.0	0	0.0	N/A		N/A		N/A	
71. Marine and fresh water animals with sauce (e.g., shrimp with lobster sauce)	40	0.3	0	0.0	N/A		N/A		N/A	
72. Marine and fresh water animals without sauce (e.g., plain or fried fish/shellfish, fish cakes) with or without breading/batter	209	1.4	2	1.0	0	0.0	2	100.0	0	0.0
73. Marine and fresh water animals, canned	129	0.9	0	0.0	N/A		N/A		N/A	
74. Marine and fresh water animals, smoked or pickled, or spreads	56	0.4	0	0.0	N/A		N/A		N/A	
10. Fruit, fruit juices	1089	7.2	58	5.3	18	31.0	40	69.0	0	0.0
75. Fruit, fresh, canned or frozen, except those listed as separate item	278	1.8	13	4.6	9	69.2	4	30.8	0	0.0
76. Candied or pickled fruit	5	0.0	0	0.0	N/A		N/A		N/A	
77. Dried fruit (e.g., raisins, dates, figs)	141	0.9	0	0.0	N/A		N/A		N/A	
78. Fruit for garnish or flavor (e.g., maraschino cherries)	5	0.0	0	0.0	N/A		N/A		N/A	
79. Fruit relishes	14	0.1	0	0.0	N/A		N/A		N/A	
81. Cranberries, lemons, limes, used as ingredients	3	0.0	0	0.0	N/A		N/A		N/A	
83. Juices, nectars and fruit drinks for use as substitutes for fruit juices	636	4.2	45	7.1	9	20.0	36	80.0	0	0.0
84. Juices, used as ingredients (e.g., lemon or lime juice)	7	0.1	0	0.0	N/A		N/A		N/A	
11. Legumes	180	1.2	0	0.0	N/A		N/A		N/A	
85. Bean curd (tofu) and tempeh	16	0.1	0	0.0	N/A		N/A		N/A	
86. Beans, peas, lentils (e.g., chickpeas, kidney beans, soybeans)	164	1.1	0	0.0	N/A		N/A		N/A	
12. Meat, poultry, their products, substitutes	895	5.9	4	0.5	1	25.0	3	75.0	0	0.0
87. Pork rinds and bacon	38	0.3	0	0.0	N/A		N/A		N/A	
88. Beef, pork and poultry breakfast strips	11	0.1	0	0.0	N/A		N/A		N/A	
89. Dried meat and poultry (e.g., jerky, Parma ham) and dry sausage products (e.g., salami)	77	0.5	0	0.0	N/A		N/A		N/A	
90. Luncheon meats (e.g., bologna, mortadella), pâté, sandwich spread, taco fillings, meat pie fillings	101	0.7	0	0.0	N/A		N/A		N/A	
91. Sausage products (e.g., wieners breakfast sausage, pepperoni, frankfurters)	172	1.1	0	0.0	N/A		N/A		N/A	
92. Cuts or meat and poultry without sauce, and ready-to-cook cuts, with or without breading/batter, including marinated, tenderized, injected cuts	123	0.8	0	0.0	N/A		N/A		N/A	
93. Patties, cutlets, choppettes, steakettes, meatballs, sausage meat, ground meat, with or without breading/batter	191	1.3	4	2.1	1	25.0	3	75.0	0	0.0
94. Cured meat products (e.g., cured ham, back bacon, corned beef, smoked meat etc.)	86	0.6	0	0.0	N/A		N/A		N/A	
95. Canned meat and poultry	23	0.2	0	0.0	N/A		N/A		N/A	
96. Meat and poultry with sauce (e.g., meat in barbeque sauce, turkey with gravy), excluding combination dishes	73	0.5	0	0.0	N/A		N/A		N/A	
13. Miscellaneous	450	3.0	14	3.1	0	0.0	8	57.1	6	42.9
97. Baking powder, baking soda, pectin	5	0.0	0	0.0	N/A		N/A		N/A	
98. Baking decorations (e.g., colored sugars, sprinkles)	6	0.0	4	66.7	0	0.0	4	100.0	0	0.0
99. Bread crumbs and batter mixes	211	1.4	4	1.9	0	0.0	4	100.0	0	0.0
101. Cocoa powder	4	0.0	0	0.0	N/A		N/A		N/A	
102. Non-alcoholic drink mixers (e.g., piña colada, daiquiri)	5	0.0	0	0.0	N/A		N/A		N/A	
104. Salad and potato toppers (e.g., salad crunchies, crispins, substitutes for bacon bits)	28	0.2	0	0.0	N/A		N/A		N/A	
105. Salt and salt substitutes, seasoned salts (e.g., garlic salt)	17	0.1	0	0.0	N/A		N/A		N/A	
106. Spices and herbs	170	1.1	6	3.5	0	0.0	0	0.0	6	100.0
14. Combination Dishes	1357	8.9	69	5.1	20	29.0	49	71.0	0	0.0
107. Measurable with a cup (e.g., casserole, macaroni and cheese, stir fry, chili, ravioli in sauce, poutine)	671	4.4	24	3.6	6	25.0	18	75.0	0	0.0
108. Not measurable with a cup (e.g., burritos, egg rolls, pizza, quiche, stuffed vegetables, shish kabobs, meat and poultry lunch packages)	522	3.4	38	7.2	11	28.9	27	71.1	0	0.0
109. Meat pie and tourtière	164	1.1	7	4.2	3	42.9	4	57.1	0	0.0
15. Nuts, seeds	220	1.4	9	4.1	4	44.4	5	55.6	0	0.0
110. Nuts and seeds, not for use as snacks, whole, chopped, sliced, slivered, ground	130	0.9	0	0.0	N/A		N/A		N/A	
111. Butters, pastes, creams (excl. peanut butter)	33	0.2	4	12.1	4	100.0	0	0.0	0	0.0
112. Peanut butter	50	0.3	5	10.0	0	0.0	5	100.0	0	0.0
113. Flours (e.g., coconut flour)	7	0.1	0	0.0	N/A		N/A		N/A	
16. Potatoes, sweet potatoes, yams	140	0.9	4	2.9	2	50.0	2	50.0	0	0.0
114. French fried, hash browns, skins and pancakes	69	0.5	4	5.8	2	50.0	2	50.0	0	0.0
115. Mashed, candied, stuffed or with sauce	56	0.4	0	0.0	N/A		N/A		N/A	
116. Plain, fresh, canned or frozen	15	0.1	0	0.0	N/A		N/A		N/A	
17. Salads	70	0.5	0	0.0	N/A		N/A		N/A	
117. Salads (e.g., Egg, fish, shellfish, bean, fruit, vegetable, meat), except those as separate item	46	0.3	0	0.0	N/A		N/A		N/A	
119. Pasta or potato salad	24	0.2	0	0.0	N/A		N/A		N/A	
18. Sauces, dips, gravies, condiments	1229	8.1	0	0.0	N/A		N/A		N/A	
120. Sauces for dipping (e.g., barbeque, hollandaise, tartar, mustard, sweet and sour)	104	0.7	0	0.0	N/A		N/A		N/A	
121. Dips (legume or dairy-based)	140	0.9	0	0.0	N/A		N/A		N/A	
122. Major main entrée sauce (e.g., spaghetti sauce)	225	1.5	0	0.0	N/A		N/A		N/A	
123. Minor main entrée sauce (e.g., pizza sauce, pesto sauce, salsa, etc.)	287	1.9	0	0.0	N/A		N/A		N/A	
124. Major condiments (e.g., ketchup, steak sauce, vinegar, marinades)	375	2.5	0	0.0	N/A		N/A		N/A	
125. Minor condiments (e.g., horseradish, hot sauce, mustard, Worcestershire sauce)	98	0.6	0	0.0	N/A		N/A		N/A	
19. Snacks (Chips, pretzels, meat or nut snacks)	794	5.2	54	6.8	10	18.5	44	81.5	0	0.0
126. Chips, pretzels, popcorn, extruded snacks, grain-based snack mixes and fruit-based snacks (e.g., fruit chips)	558	3.7	52	9.3	10	19.2	42	80.8	0	0.0
127. Nuts or seeds for use as snacks	223	1.5	2	0.9	0	0.0	2	100.0	0	0.0
128. Meat or poultry snack food sticks	13	0.1	0	0.0	N/A		N/A		N/A	
20. Soups	456	3.0	1	0.2	0	0.0	1	100.0	0	0.0
129. All varieties	456	3.0	1	0.2	0	0.0	1	100.0	0	0.0
21. Sugars, sweets	749	4.9	72	9.6	0	0.0	62	86.1	10	13.9
130. Candies (incl. chocolate bars and chocolate products), except those as separate item	377	2.5	45	11.9	0	0.0	45	100.0	0	0.0
131. Hard candies, except those as separate item	8	0.1	3	37.5	0	0.0	3	100.0	0	0.0
132. Baking candies, such as chocolate chips	30	0.2	1	3.3	0	0.0	1	100.0	0	0.0
133. Breath mints	6	0.0	0	0.0	N/A		N/A		N/A	
135. Confectioner’s or icing sugar	1	0.0	0	0.0	N/A		N/A		N/A	
136. Bread spreads, except those as separate item, honey, molasses	50	0.3	9	18.0	0	0.0	0	0.0	9	100.0
137. Jams, jellies, marmalades, fruit butters, spreads	193	1.3	1	0.5	0	0.0	0	0.0	1	100.0
138. Marshmallows	13	0.1	3	23.1	0	0.0	3	100.0	0	0.0
139. Sugars, except those as separate item	6	0.0	0	0.0	N/A		N/A		N/A	
140. Sugar substitute	3	0.0	0	0.0	N/A		N/A		N/A	
141. Syrups (incl. chocolate, maple and corn syrup)	62	0.4	10	16.1	0	0.0	10	100.0	0	0.0
22. Vegetables	834	5.5	0	0.0	N/A		N/A		N/A	
142. Vegetables without sauce (incl. cream style corn, stewed tomatoes), except vegetables without sauce listed as separate item	418	2.7	0	0.0	N/A		N/A		N/A	
143. Vegetables with sauce	17	0.1	0	0.0	N/A		N/A		N/A	
144. Vegetables for use as garnish/flavoring, fresh, canned or frozen, but not dried (e.g., parsley or garlic)	24	0.2	0	0.0	N/A		N/A		N/A	
145. Chili pepper and green onion	42	0.3	0	0.0	N/A		N/A		N/A	
146. Seaweed	6	0.0	0	0.0	N/A		N/A		N/A	
147. Lettuce and spouts	17	0.1	0	0.0	N/A		N/A		N/A	
148. Vegetable juice and vegetable drink	43	0.3	0	0.0	N/A		N/A		N/A	
149. Olives	83	0.5	0	0.0	N/A		N/A		N/A	
150. Pickles	112	0.7	0	0.0	N/A		N/A		N/A	
151. Relish	24	0.2	0	0.0	N/A		N/A		N/A	
152. Vegetable pastes, (e.g., tomato paste)	12	0.1	0	0.0	N/A		N/A		N/A	
153. Vegetable sauce or puree (e.g., tomato sauce or tomato puree)	36	0.2	0	0.0	N/A		N/A		N/A	

^1^ Packaged food products are from the Food Label Information Program (FLIP) 2013, as described in [22]. ^2^ Products with child-directed packaging were identified using criteria based on previous publications [23]. ^3^ Canadian Children’s Food and Beverage Advertising Initiative’s (CAI) Uniform Nutrition Criteria are defined in: Advertising Standards Canada. *Canadian Children’s Food and Beverage Advertising Initiative*; Uniform Nutrition Criteria White Paper; September 2014 [17]. ^4^ Food categories are defined in Schedule M of the Food and Drug Regulations (C.R.C., c. 870), Sections B.01.001, B.01.002A and D.01.001, Government of Canada, Health Canada [21]. ^5^ Percentage of total products (i.e., out of *n* = 15,231 products). ^6^ Percentage of total products in that food category. ^7^ Percentage of products with child-directed packaging in that food category.
